# *SMPD1* expression profile and mutation landscape help decipher genotype–phenotype association and precision diagnosis for acid sphingomyelinase deficiency

**DOI:** 10.1186/s41065-023-00272-1

**Published:** 2023-03-13

**Authors:** Ruisong Wang, Ziyi Qin, Long Huang, Huiling Luo, Han Peng, Xinyu Zhou, Zhixiang Zhao, Mingyao Liu, Pinhong Yang, Tieliu Shi

**Affiliations:** 1 College of Life and Environmental Sciences, Hunan University of Arts and Science, 3150 Dongting Ave., Changde, 415000 Hunan Province People’s Republic of China; 2 Affiliated Hospital of Hunan University of Arts and Science (the Maternal and Child Health Hospital), Medical college, 3150 Dongting Ave., Changde, Hunan Province People’s Republic of China 415000; 3 Changde Research Centre for Artificial Intelligence and Biomedicine, 3150 Dongting Ave., Changde, 415000 Hunan Province People’s Republic of China

**Keywords:** Acid sphingomyelinase deficiency, Niemann-pick disease type a and B, Genotype, Phenotype, Novel target for the subtypes

## Abstract

**Background:**

Acid sphingomyelinase deficiency (ASMD) disorder, also known as Niemann–Pick disease (NPD) is a rare genetic disease caused by mutations in *SMPD1* gene, which encodes sphingomyelin phosphodiesterase (ASM). Except for liver and spleen enlargement and lung disease, two subtypes (Type A and B) of NDP have different onset times, survival times, ASM activities, and neurological abnormalities. To comprehensively explore NPD’s genotype-phenotype association and pathophysiological characteristics, we collected 144 NPD cases with strict quality control through literature mining.

**Results:**

The difference in ASM activity can differentiate NPD type A from other subtypes, with the ratio of ASM activity to the reference values being lower in type A (threshold 0.045 (4.45%)). Severe variations, such as deletion and insertion, can cause complete loss of ASM function, leading to type A, whereas relatively mild missense mutations generally result in type B. Among reported mutations, the p.Arg3AlafsX76 mutation is highly prevalent in the Chinese population, and the p.R608del mutation is common in Mediterranean countries. The expression profiles of *SMPD1* from GTEx and single-cell RNA sequencing data of multiple fetal tissues showed that high expressions of *SMPD1* can be observed in the liver, spleen, and brain tissues of adults and hepatoblasts, hematopoietic stem cells, STC2_TLX1-positive cells, mesothelial cells of the spleen, vascular endothelial cells of the cerebellum and the cerebrum of fetuses, indicating that SMPD1 dysfunction is highly likely to have a significant effect on the function of those cell types during development and the clinicians need pay attention to these organs or tissues as well during diagnosis. In addition, we also predicted 21 new pathogenic mutations in the *SMPD1* gene that potentially cause the NPD, signifying that more rare cases will be detected with those mutations in *SMPD1*. Finally, we also analysed the function of the NPD type A cells following the extracellular milieu.

**Conclusions:**

Our study is the first to elucidate the effects of *SMPD1* mutation on cell types and at the tissue level, which provides new insights into the genotype-phenotype association and can help in the precise diagnosis of NPD.

**Supplementary Information:**

The online version contains supplementary material available at 10.1186/s41065-023-00272-1.

## Background

Lysosome storage disease (LSD) is a collection of inherited metabolic illnesses. There are about 50 classified LSDs [1, 2]. LSD occurs when lysosomes are unable to degrade macromolecules, such as fats and sugars, and the then deposition of macromolecules to toxic levels in organelles forms cell inclusions, which causes various signs and symptoms. Gaucher’s disease (GD), Krabbe disease (KD), Metachromatic Leukodystrophy (ML) and Niemann-Pick disease (NPD) are all inherited rare autosomal recessive LSDs resulting from impaired degradation of sphingolipids with different deficiency of the upstream enzymes [2]. GD results from the deficiency of the enzyme *β*-glucosidase [3–6]. Genetic mutations in *GALC* result in a deficiency of galactosylceramidase, leading to KD [1] which was first described one [7]. Abnormal functions of arylsulfatase resulting from the mutations in the *ARSA* and *PSAP* genes (more rarely) [8] lead to ML. NPD types A and B are due to a deficiency of the enzyme sphingomyelinase [3, 9].

Recognising it as a spectrum of disorders, the nomenclature NPD is no longer used in some specific areas; instead, the disorder is called acid sphingomyelinase deficiency (ASMD). However, recent studies still use Niemann-Pick disease for clarity. In patients with hepatosplenomegaly, lipid-filled foam-like cells can be seen in the bone marrow, brain, and organs often called Niemann–Pick cells. The cause for NPD type A (NPA, MIM257200) and NPD type B (NPB, MIM607616) have been clarified with the mutations in the *SMPD1* gene encoding sphingomyelin phosphodiesterase-1 [10] after ASMD was reconginzed as the factor for type A NPD [11] . Thus, patients with NPA and NPB can be diagnosed with the measurement of acid sphingomyelinase (ASM). NPD type C (NPD-C1, MIM257220; or NPD-C2, MIM601015) results from a defect in the transport of low-density lipoprotein cholesterol in cells [12, 13]. Type D is also known as Nova Scotia NPD; some cases are an allelic variant of NPD-C1 [14, 15]. However, type D NPD is no longer used [10].

The *SMPD1* gene comprises six exons and spans 5 kb on chromosome 11p15.4-p15.1 [16]. It encodes the human ASM protein (UniProt ID P17405) with 631-amino acids composed of a saposin domain, a proline-rich linker, a metallophosphatase catalytic domain, and a C-terminal domain. Six potential N-linked glycosylation sites, eight disulfides, and two zinc ions in ASM play critical roles in protein folding and stability [17, 18]. By maintaining proper sphingolipid homeostasis and participating in membrane turnover, ASM interacts with other lipid hydrolases within lysosomes [10]. During stress, ASM translocates rapidly from lysosomes to the plasma membrane and hydrolyses sphingomyelin into ceramide [19, 20]. As a result, membrane lipid microdomains, or “rafts,” are reorganised, stimulating downstream signalling [10].

Symptoms of NPA include central nervous system (CNS) deterioration, cherry-red macula, and massive hepatosplenomegaly, leading to death at an early age, whereas patients with NPB have a better prognosis [21], which symptoms are non-neuropathic. Patients with NPA often die before diagnosis (early onset) [22]. Most NPB patients could survive into adulthood and even until the 70s. Currently, NPA and NPB have no efficient treatment. Bone marrow transplantation [23] and liver transplants [24] have been undertaken in NPD patients, but these treatments could not cure the disease. FDA has granted Enzyme Replacement Therapy, and now its effectiveness is evaluating. It appears, however, that this treatment is unlikely to have a profound effect on the disease’s neurological features [10].

Since the pathogenic factor for the two NPD types are mutations in the *SMPD1* gene, systematically exploring the underlying mechanism that causes these two subtypes is necessary. Because the pathogenic mutations of the *SMPD1* gene are primarily found in compound heterozygotes,investigating the phenotype-genotype association is the key to distinguishing the two subtypes [21, 22, 25, 26], hence, to enable families to understand better how the diagnosis affects their children’s health and well-being. Genotype-phenotype relationships for pathogenicity could be associated with the molecular basis, such as gene mutation and expression [27]. Therefore, it is hypothesised that various mutation sites of the *SMPD1* gene and its expressions in various cells are associated with early-onset and late-onset phenotypes. Comprehensive analyses would help understand the mechanisms underlying the phenotypes [28] and information for the therapies. Therefore, in this study, we collected clinical cases reported in the literature and illustrated the landscape of mutations in *SMPD1* to explore how those mutations affect the protein function, including the physical and chemical properties of the protein and ASM activity. In addition, we also applied several algorithms to predict variants that potentially cause dysfunction of the *SMPD1* gene and result in NPD.

## Results

### Case collection

To thoroughly investigate the genotype-phenotype association in NPA and NPB, reported cases were extracted from the PubMed database through a manual curation process. A total of 144 cases with mutation information and additional 23 cases reporting ASM levels but lack of detailed mutation information of the patients were collected (the detailed information can be found in Supplementary Table S1), and the following data were extracted: onset age, sex, ethnicity, mutation site of *SMPD1* gene, clinical phenotypes and symptoms, and ASM activity level for each case based on the standardised format and terms.

### ASM activity level in the collected clinical cases

**Fig. 1:**
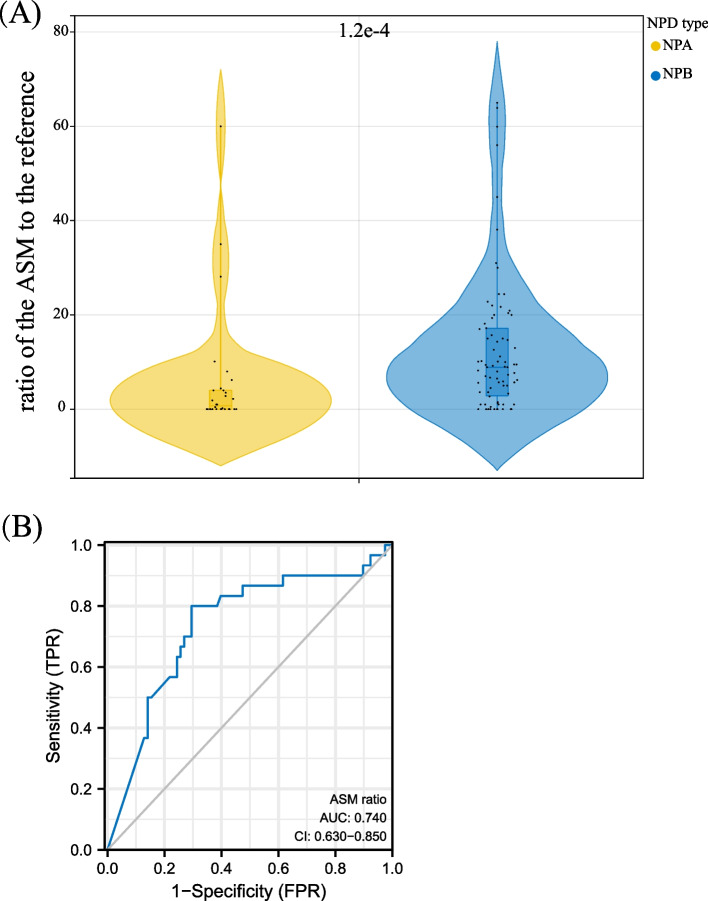
A novel threshold for determining NPD subtypes. **A** ASM levels in clinical cases are collected. **B** ROC curve for the predictability of the threshold. ASM ratio, the ratio of the activity of acid sphingomyelin phosphodiesterase of the patients to the reference value; NPA, Niemann–Pick disease type A; NPB, Niemann–Pick disease type B; ROC, receiver operating characteristic curve; AUC, area under the curve

Currently, no conclusive criteria exist to identify NPA and NPB at the physiological and biochemical levels. A previous study reported that the ASM level in plasma could be used as an index to differentiate NPA from NPB in Chinese populations [29]. To test whether the ASM level in plasma as an index can be extended to other populations, we analysed the ASM levels in collected cases to find the threshold value to differentiate NPA from NPB based on the ratio of the ASM activity in patients to the reference constructed with healthy people (or normal ASM enzymes). When excluding the Chinese cases for this analysis, we then collected additional papers consisting of 23 non-Chinese cases (most of them are clinical reports) that reported ASM levels (in Supplementary Table S2). These additional papers are not used for phenotype-genotype correlation due to a lack of mutation information. Thus, 108 cases were included. As a result, NPA and NPB demonstrated a significant difference in ASM activities by t-test (p = 1.2 × 10^−4^, Fig. 1A), but no significant difference was found between the intermediate NPD and NPB. NPA can be differentiated from other subtypes (NPB and the intermediate group) at a threshold of 4.45% of ASM activity with an AUC value of 0.740, the Sensitivity of 0.800, a Specificity of 0.705, and Youden’s index of 0.505 (Fig. 1B) indicating that patients without neurological involvement normally have the ASM residual activity-to-control ratio over 0.045 (4.45%).

### Mutations in the clinical cases collected

**Fig. 2:**
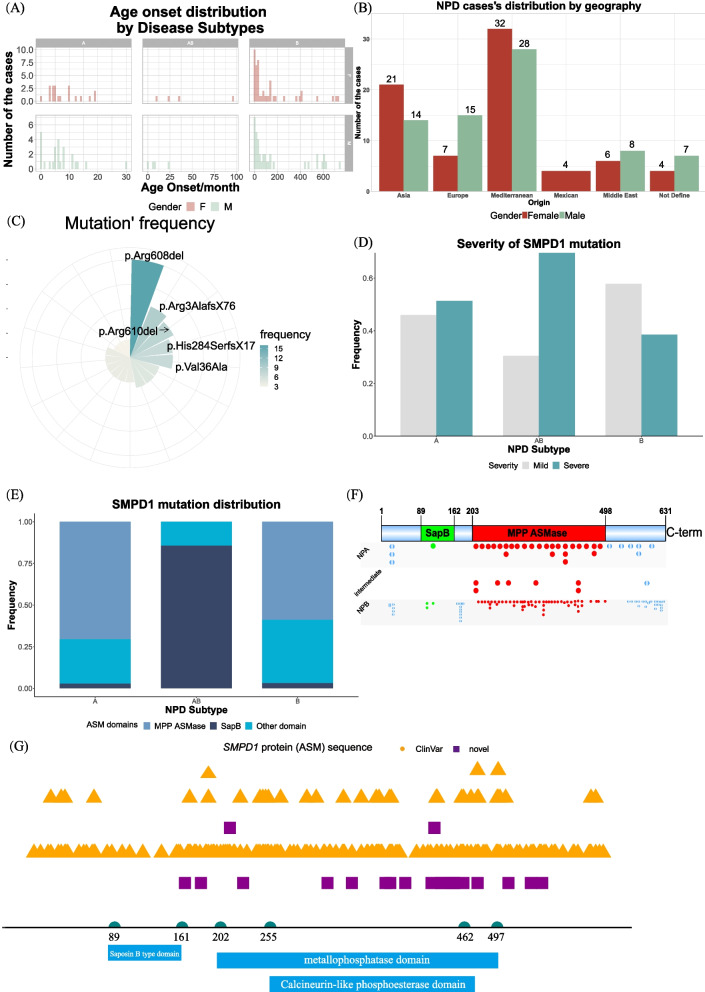
Statistics of the mutation sites on the *SMPD1* gene. **A** Distribution of the patients by onset age. F, female. M, male. **B** Country of origin of the patients: Mediterranean countries, including Italy, Algeria, Spain, Turkey, Maghreb, Jordan, and North Africa; Asian countries, such as China and Japan; European countries, such as Caucasian (documented by the research), Poland, Gypsy (documented by the research), and the Netherlands; Middle East countries including Iran and Palestine. **C** Amino acid mutation frequency in all collected cases. **D** *SMPD1* mutation types (do not include all mutations). Severe mutations include deletions, insertions, and nonsense. **E** *SMPD1* mutation distribution in domains of ASM domains in each subtype of NPD. A, NPA. AB, intermediate NPD subtype. B, NPB. **F** Distribution of missense mutations on the conserved domain of the human ASM protein. Each point represents one reported mutation in the collected cases. Points are coloured according to the domains. **G** The landscape of *SMPD1* mutations and 21 novel pathogenic variants prediction based on the databases of ClinVar, ANNOVAR and the EVE model. Purple squares, novel predicted pathogenic variants. Orange triangles, pathogenic/likely pathogenic variants from the ClinVar. Domains were annotated by NCBI. Please note: in **A**, **E** panels, A, NPA. AB, intermediate. B, NPB. In **D**, **F**, and **G**, domains were retrieved from the NCBI (NP_000534.3), namely, saposin (B) (smart00741, Location: 89 → 161), metallophosphatase domain (cd00842, Location: 202 → 497) and Calcineurin-like phosphoesterase domain (Metallophos for short, pfam00149, Location: 255 → 462)

Normally, patients with NPA have high pathogenicity mutations in *SMPD1*, with early disease onset. Previous studies have demonstrated that patients with diverse NPD subtypes have significantly different severities [29, 30]. In the present study, we collected 144 cases and showed that the onset age for most patients with NPA was between 0 and 10 months (less than 1 year), while the onset age of patients with NPB was aggregated between 0 and 200 months (16.7 years) (Fig. 2A). Among those patients, sixty patients with NPD lived in Mediterranean countries, including Italy, Algeria, Spain, Turkey, Maghreb, Jordan, and North Africa. All 22 patients from Europe were Caucasians, Polish, Gypsy or Dutch. Thirty-five patients with NPD have Asian backgrounds (China and Japan) (Fig. 2B). In the cases collected, there were more patients with NPD from the Mediterranean area, although the results might be biased during the case collection from PubMed. From these cases, the most common mutation is p.Arg608del, followed by p.Arg3AlafsX76 and p.Arg610del (Fig. 2C).

It has been noticed that different mutation types would lead to distinct phenotypes of NPD; therefore, the different types of mutations were annotated (Fig. 2D). Duplications, nonsense mutation, deletions and insertions are considered severe mutations, while missenses are mild to severe ones. About 50% of the mutations belong to the severe type in NPA in the collected cases, a higher prevalence than that in NPB (42% roughly). The mild mutations counted for 57% of all mutations in NPB, higher than the frequency in NPA. Of all the annotated missense mutations, over 70% occurred in the metallophosphatase domain, while only a tiny portion of missense mutation happened in Saposin (B) Domains (Fig. 2E&F).

### Pathogenicity at different sites in *SMPD1*

ANNOVAR is a software tool that utilises genetic and evolutionary information to annotate genetic variants detected from diverse genomes functionally. A total of 1203 variants of the *SMPD1* gene have been annotated in ANNOVAR. We retrieved their corresponding SIFT_score, Polyphen2_HVAR _score, Polyphen2_HDIV_score, MutationTaster, M-CAP score and CADD_Phred. We also collected the annotation information for *SMPD1* variants from the ClinVar database; 591 variants in the ClinVar and 203 pathogenic/likely pathogenic variants in exon regions were filtered and mapped in the diagrammatic sketch (Fig. 2G). There are 44 variants annotated in the ANNOVAR but not the ClinVar database. Following the pathogenicity threshold defined by their corresponding authors (SIFT_score < 0.05, Polyphen2_HDIV_score > =0.957, MetaLR_score > 0.5, CADD_phred> 20, and M-CAP_score > 0.025 and disease_causing labelled by MutationTaster_pred), 38 of the 44 variants are annotated as pathogenicity (Supplementary Table S3), but none of them has been reported in literature. In addition, eight variants, including c.G491T, c.G1026T, c.C1279A, c.C1288G, c.C1288T, c.T1309C, c.A1351C and c.A1382C, were not found in the gnomAD and other variants frequency were relatively low, only 3 (c.G394T, c.C995A, and c.C1598A) were annotated in Han Chinese people (Huabiao), which indicated the population-specific pattern for some mutations.

Next, we adapted a deep learning algorithm, the EVE model, to further predict the clinical significance of those mutations in *SMPD1* gene. EVE is a new method to predict the clinical significance of human variants based on sequences of diverse organisms across evolution [30]. With the EVE model, 23 of the 44 variants were predicted to be pathogenic. Among the 44 variants, 21 were predicted to be pathogenic with both methods (their locations are shown in Fig. 2G roughly).

### Differences in the distribution of variant sites among different ethnicities

**Fig. 3:**
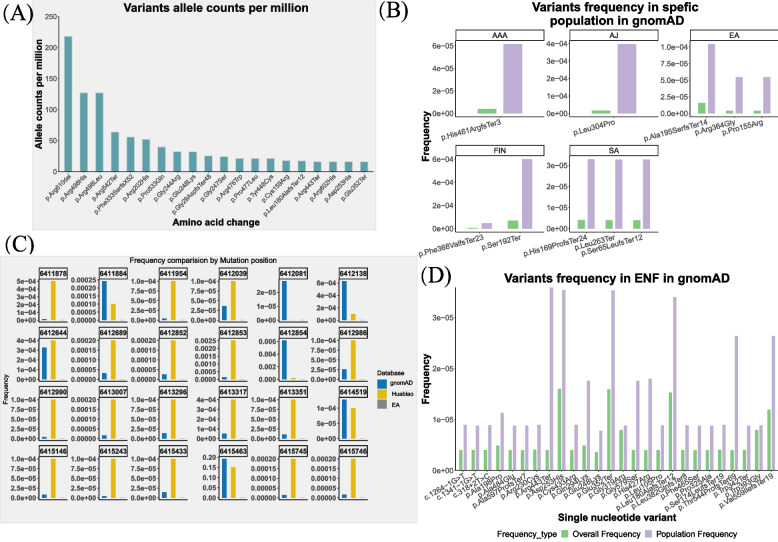
Pathogenic variants allele distribution. **A** Top 20 pathogenic variants in gnomAD. **B** Frequency of the pathogenic/likely pathogenic variants with population specificity in gnomAD. AAA, African/African American. AJ, Ashkenazi Jewish. EA, East Asian. ENF, European (non-Finnish). FIN, Finnish. LAA, Latino/Admixed American. SA, South Asian. **C** *SMPD1* mutation allele frequency in two data sources. Huabiao, the public project database of whole exomes of the Chinese Han. EA, data from east Asia of the gnomAD

To survey whether the mutation profile in the *SMPD1* gene has any ethnic prevalence, we first plotted the top 20 variants that have been annotated as pathogenic/likely pathogenic ones according to their allele frequency (Fig. 3A). Among 36 pathogenic/likely pathogenic mutations detected in gnomAD (Fig. 3B), p.Arg610del has been found in nearly all ethnicities. The variants at the 498th amino acid (missense and changed to His or Leu), namely p.Arg498His or p.Arg498Leu, share the second place. p.His461ArgfsTer3 was found specific to African/African Americans. The Jewish people carry a unique variant, p.Leu304Pro, while p.Ala195SerfsTer14, p.Arg364Gly and p.Pro155Arg are the three variants specifically reported in East Asians. There are two unique mutations in Finnish populations. South Asians carry three protein changes induced by single nucleotide variants.

We further explored whether any significant difference could be observed between the Han Chinese group and other East Asians. Comparing gnomAD and Huabiao databases, we have found that the allele frequency of 24 variants was differently recorded (Fig. 3C). For example, the variants occurring at sites of 6,411,878 (p.Arg17Gln or p.Arg17Pro), 6,411,954 (p.Ala44_Leu49del) and 6,415,243 (p.Ser486Arg) in gnomAD were nearly 0, but in Huabiao, they were more frequent. In contrast, the frequency at the mutation sites of 6,412,081 and 6,412,854 was much higher than that of the variants in the Huabiao. Additionally, in the East Asian people (the subtype of the gnomAD), the 24 mutation sites have not been included.

### Phenotype-genotype correlation

In this analysis, 144 cases with correspondingly detailed documentation of the complete mutation information were utilised.

#### Different variations are associated with different phenotypes

The amino acid glycine at site 247, located in the conserved metallophosphatase domain, is a highly prevalent mutation site affected by nucleotide changes of c.740delG, c.741delG, and c.739G > A in 7 patients. Variations at this site have been associated with NPA [31–33]. Patients hosting the deletions (c.740delG or c.741delG) and c.739G > A show disease onset age less than 6 months and died by the age 3 years old, which result in a global developmental delay, seizure, psychosis, and other nervous system-related diseases, together with the liver- and spleen-related symptoms. In contrast, patients with most missense mutations at glycine site 247 were not diagnosed with NPA but NPB (mean onset age: 45.6 ± 10.9 years)., except c.739G > A and c.1159 T > C (p.[Cys387Arg]). Similarly, patients with homozygous or heterozygous variants c.1828_1830delCGC (p.Arg610del) were all associated with NPB without nervous system involvement.

#### Recurrent variants of c.4delC (p.Arg3AlafsX76) and c.842-849dup8 (p.His284SerfsX17) in Chinese origin correlates with NPA/B or the intermediate

Among all 28 reported variants in our collection, c.4delC (p.Arg3AlafsX76) and c.842-849dup8 (p.His284SerfsX17) in Chinese patients have high prevalence [34]. The duplication variant occurs in six patients (mean onset age: 2.5 ± 1.6 months) with seven alleles. The mean of ASM activities was significantly low (5.5 ± 0.67% to the reference). Psychomotor regression and hypotonia were the main phenotypes related to the nervous system of patients with the mutation. For deletion of c.4delC (p.Arg3AlafsX76), nine alleles were detected in six patients. Although the deletion variants can cause severe ASM dysfunction, Chinese patients with one variant on the alleles (heterozygous) were diagnosed with the intermediate type, indicating haploinsufficiency, while patients with homozygous alleles (two c.4delC on the alleles) were diagnosed with NPB. This gene mutation appeared non-neurotoxic as the detected ASM activity was relatively high (*mean* 25 %  ± 0.56%). Thus, the two variants mentioned above are associated with the discrimination of the NPD.

#### Variants of c.1823_1825delCCG (p.R608del) correlate with the NPB phenotype

Among our collected cases, 14 patients had at least one *SMPD1* p.R608del allele variant (homozygous, *n* = 9; heterozygous, *n* = 5) associated with NPB clinical phenotypes [35–37]. Interestingly, 13 of these patients were reported to live in Mediterranean countries (Italy, Algeria, Spain, Turkey, etc.). This variant has not been reported from people living in other areas except America. Nearly all patients with homozygous or heterozygous mutations survive to adulthood, with the oldest patient 60 years old. None of them was reported to have neurological diseases, and most of them had active ASM. Thus, c.1823_1825delCCG (p.R608del) variants are primarily associated with NPB in Mediterranean patients, and the deletion of amino acid proline has a minor impact on the patients since it happens near the C-terminal of the protein.

### The expression pattern of the *SMPD1* gene

Gene performs its functions only in the cells/tissues it expresses. To comprehensively explore the tissues affected by *SMPD1* gene mutations, we collected the expression pattern of the *SMPD1* gene from the GTEx portal and Descartes database. GTEx is a data resource and tissue bank that currently includes approximately 11,688 RNA-seq samples across 53 tissue sites, and the Descartes database contains the gene expressions of over 4 million cells of 121 human tissues during development. Since hepatosplenomegaly and splenomegaly are the most commonly observed clinical syndrome of NPB and higher incidence of neuronopathy with rapid progressive psychomotor deterioration are reported in NPA, we mainly focused on the expression patterns of *SMPD1* in the brain, liver, and spleen.

**Fig. 4:**
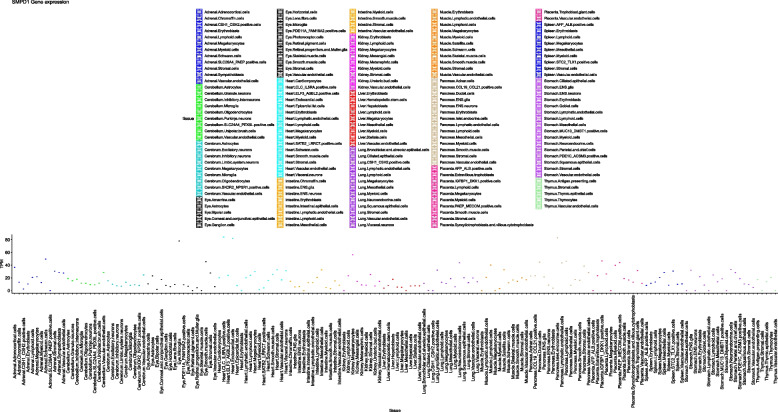
*SMPD1* gene expression in all tissues. Expression profiles of the *SMPD1* gene in different organs based on the GSE156793

Considering that NPA is the most severe clinical form with early-onset CNS involvement, we believed that dysfunctions caused by pathogenic mutations of the *SMPD1* gene could significantly affect fetal development and functions of related organs. Therefore, we evaluated the expression patterns of the *SMPD1* gene in various fetal tissues from the Descartes database (Fig. 4). The single-cell dataset of fetuses in the Descartes database is generated from 121 human fetal samples of 72–129 days in estimated postconceptual age and represents 15 organs in Fig. 4 [38]. As a result, we observed that the *SMPD1* gene is nearly expressed in all studied fetal organs, and the highest expression level is found in CLC_IL5RA-positive cells of the heart, followed by endocrine cells in pancreatic islets, endocardial cells, retinal microglia, and megakaryocytes in the kidney in Fig. 4. The expression pattern of *SMPD1* in these organs indicates that SMPD1 dysfunction should significantly affect the functions and development of the heart, pancreatic islets, eyes, and kidneys. The highest expression level of the *SMPD1* gene in liver is detected in hepatoblast cells, followed by hematopoietic stem cells. In the spleen, the highest expression level was observed in STC2_TLX1-positive cells, followed by mesothelial cells. In the brain, the highest expression level was found in the vascular endothelial cells of the cerebellum, followed by the vascular endothelial cells of the cerebrum and astrocytes of the cerebellum. In the cerebellum, *SMPD1* presented a relatively low expression profile in astrocytes, Purkinje neurons, SLC24A4_PEX5L-positive cells, oligodendrocytes, and microglia. A similar expression profile was observed for *SMPD1* in inhibitory neurons, astrocytes, limbic system neurons, oligodendrocytes and microglia of the cerebrum. In terms of protein expression, in the female postnatal brain, SPMD1 protein is expressed the most in the medial dorsal nucleus of the thalamus, followed by the hippocampus and primary visual cortex [39] in Fig. 6B. Interestingly, *SMPD1* was highly expressed in the placenta; the highest expression level was found in IGFBP1_DKK1-positive cells, followed by PAEP_MECOM-positive cells and myeloid cells.

**Fig. 5:**
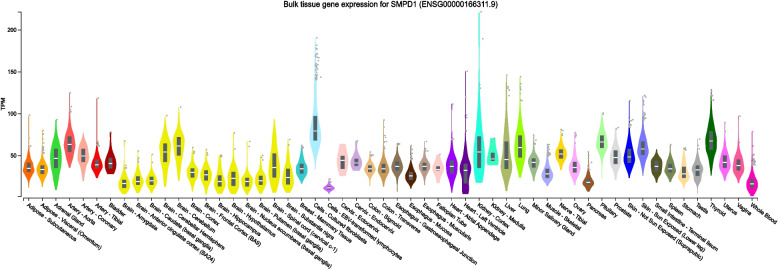
Expression profiles of the *SMPD1* gene based on GTEx data

**Fig. 6:**
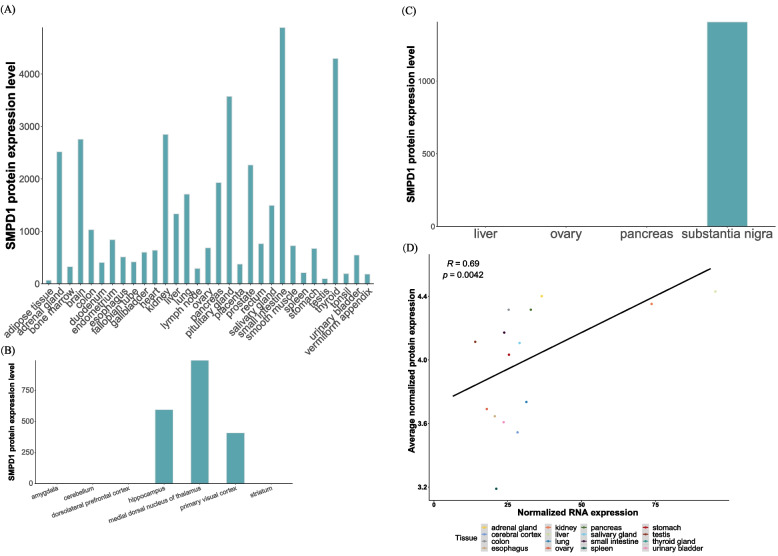
Expression profile of the SMPD1 protein with the help of the public datasets. **A** SMPD1 protein expression in Wang et al. **B** SMPD1 protein expression in the study of Carlyle et al. **C** SMPD1 protein expression in the study of Kushner et al. **D** Correlation analysis between the ASM expression at both protein and gene level by spearman. Tissues studied were the consensus with GTEx and ProteomicsDB

According to the expression patterns of the *SMPD1* gene in various tissues from GTEx (Fig. 5), thyroid tissue was found to have the highest expression of *SMPD1*, followed by the pituitary, aorta, cerebellum, lungs, skin exposed to the sun, cerebellar hemisphere, kidney cortex, tibial nerve, and coronary artery. At the protein level, the thyroid ranked 2nd in all studied tissues (Fig. 6A). The protein also demonstrated higher expression in the cerebellum, lungs, and kidney than in other tissues. In Fig. 5, the liver is ranked 13th among 53 tissues, while the spleen is ranked 20th. These two organs were all found with the expression at the protein level. Kushner et al. [40] retrospectively analysed proteomics data and found SMPD1 expression aggregated in the region of substantia nigra Fig. 6C. Then, the tissues studied in both GTEx and ProteomicsDB were retrieved and their correlation between between ASM expression at both protein and gene level were significant (r = 0.69, *p* < 0.05, Fig. 6D). The correlation indicates that transcriptome profiles would be a tool for understanding the pathogenic mechanisms underlying NPA and NPB.

**Fig. 7:**
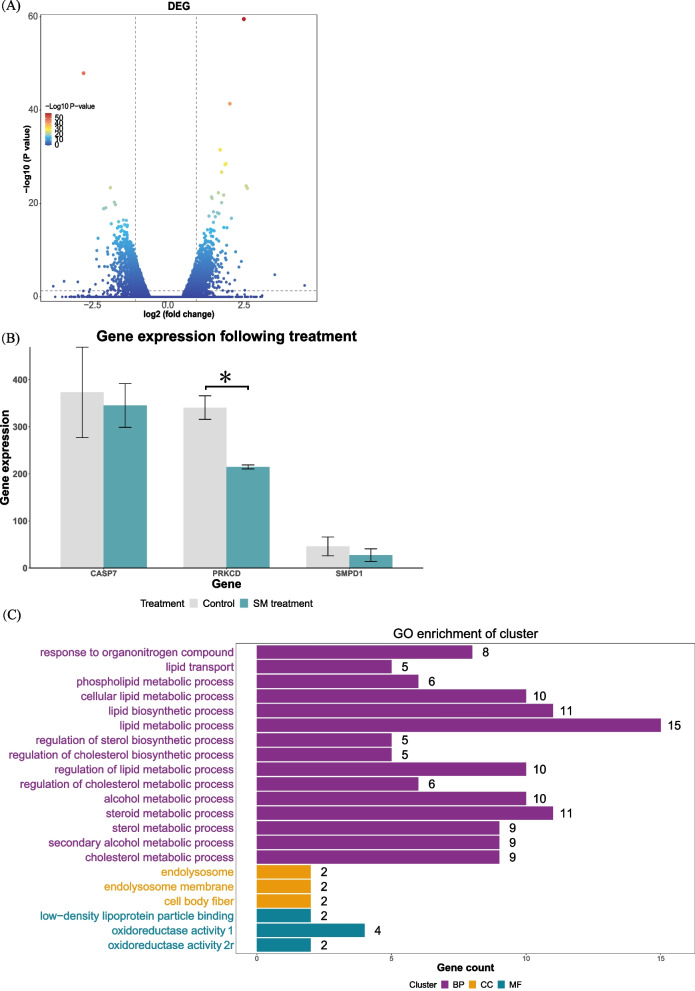
Pathways involved in NPA fibroblasts cells in response to sphingomyelin treatment. **A** DEGs illustrated with a volcano plot. **B** *CASP7*, *PRKCD* and *SMPD1* expression following the SM treatment. *, *p* < 0.05. **C** Enrichment analysis of Gene Ontology (GO) terms withtop 50 DEGS. Oxidoreductase activity 1, oxidoreductase activity, acting on paired donors, with incorporation or reduction of molecular oxygen. Oxidoreductase activity 2, oxidoreductase activity, acting on paired donors, with oxidation of a pair of donors resulting in the reduction of molecular oxygen to two molecules of water. BP, biological process. CC, cellular component. MF, molecular function

To explore the pathways when the SMPD1 deficient fibroblasts were under stress, we retrieved a dataset that the mRNAs of NPA patients’ cells were profiled, where the cells had been treated with sphingolipid sphingomyelin (SM) for 30 days compared to the control group. Figure 7A illustrates the differentially expressed genes (DEGs) (the treated cells vs the control cells).

Upon cleavage by CASP7 in the extracellular milieu, the active form is generated [41]. Stress-induced phosphorylation of Ser-510, required for secretion, is catalyzed by PRKCD protein [42, 43]. Therefore, we explored *CASP7*, *PRKCD* and *SMPD1* expression following the SM treatment (Fig. 7B). *PRKCD* expression was dropped significantly, indicating the phosphorylation of Ser-510 were not active following the SM treatment. Based on the function analysis with the top 50 DEGs (Log_2_FoldChange > 2 & FDR < 0.01) (Fig. 7C), it showed that following the treatment, the metabolic process and the regulation of lipid and alcohol were active, and endolysosome was involved when the cells were under stress (response to organonitrogen compound).

## Discussion

In the present study, we tried to comprehensively explore the association of phenotype-genotype in NPD and lay the foundation for understanding the mechanisms of this rare disease. With the strict quality-controlled literature search, we collected 144 cases with comprehensive pathophysiological characteristics of NPD. We also found a connection between some variants and the phenotypes (NPD type A or B). Type A is correlated with more severe mutations, while patients with the non-neuro-related type NPB normally have mutations in *SMPD1* with a mild effect. In addition, following the model, we have found a threshold of 4.45% that it can be taken into account to discriminate the majority of cases with NPA from clinical phenotypes less severe of NPD (intermediate and NPB). Furthermore, we also explored the expression landscape of the *SMPD1* in different cell types of fetal development and adult tissues, which offered us the opportunities to better understand pathogenic mechanisms underlying NPA and NPB at a single cell type level. At the same time, the difference in *SMPD1* expression levels on different cell types provides an important resource for the precise diagnosis of the disease in clinical application.

The ASM protein, encoded by *SMPD1* gene, catalyses the degradation of sphingomyelin into ceramide and phosphocholine [42, 44]. Following the *SMPD1* gene’s transcription, in the endoplasmic reticulum, an inactive 75-kDa pre-pro-polypeptide of 631 residues is synthesised from the 1896-bp open reading frame of the full-length human *SMPD1* cDNA [45]. It has been found that in the extracellular milieu, more SMPD1 proteins should have been produced, but the expression of *SMPD1* was not correspondingly changed under SM stress. *CASP7* gene’ encoding a protein that cleaves SMPD1 [41], expression is not significantly changed either. Here, we indeed observe significant drop of *PRKCD* expression, its protein, PKC *δ*, is required for phosphorylating SMPD1 protein for its secretion. Zeidan and Hannun suggested the existence of a positive feedback loop between PKC *δ* production and downstream ceramide formation [43]. Since the residual SMPD1 proteins still can convert SM into ceramide and ceramide metabolized by other pathways, a reduced expression of *PRKCD* appeared to be reasonable. One form of SMPD1 proteins targets the endolysosomal compartment; the other is released extracellularly [42, 44]. In normal state, as a result of signal peptide cleavage, the precursor undergoes processing in the Endoplasmic Reticulum/Golgi complex to produce a catalytic form of 70 kDa entering endosome/lysosome [45]. The extracellularly released SMPD1 proteins in the form of lysosomal exocytosis as the primary source under specific stimulations and circumstances [46, 47]. This notion correlates with the results that the endolysosome and its membrane also participate in the process (Fig. 7C). Within the data of GO enrichment, the response to the organonitrogen compound was also found, when the NPA cells were treated with SM. As suggested, SMPD1 proteins also facilitates cholesterol [48]; therefore, it has been found that regulation and metabolic processes of alcohol, lipid and cholesterol were also involved in the cells under SM stress. These pathways together would participate into relieving the toxic effects of accumulation of SM, although the complete functions of SMPD1 protein remains to be investigated.

The residual ASM activity has been regarded as one of the clinical features to distinguish NPA from NPB in Chinese people [29], while some bibliographic data that supports ASM residual activity threshold is not definitive for discriminating between type A and B [49–51]. Commonly, < 5% of effective residual ASM activity in situ is observed in NPA, whereas 5–20% is detected in NPB [22, 52–54]. However, in the literature we have mined, > 5% of the cases were still diagnosed as NPA [35, 55, 56]. Several intermediate cases indicated that the residual activities of the ASM enzyme were broad [49, 57]. Therefore, the ASM activity is not always associated with the so-called well-defined subtypes. Our model indicated that 4.45% of ASM activity could be the threshold to distinguish NPA from other subtypes. However, Hu et al. suggested that the cutoff value for differentiating the two clinical forms was 1.685 nmol/17 h/mg protein (approximately 12.2% to the reference, 13.7 nmol/17 h/mg protein as the reference value in Chinese patients only, and all ASM activities were measured within single laboratory using the same method) [29]. Although the *SMPD1* gene sequencing appears to be a golden standard for NPD diagnosis, which should not be used as a first-line indicator [10], the method might be less available in less-developed regions. It is still recommended to examine ASM activity as a standard for deficiency diagnosis [58]. The threshold proposed in the present study was derived from multinational samples (i.e., leukocytes, skin fibroblasts, or dried blood spots) and different ethnic groups [59] with different measurement approaches to ASM activity, which further indicates that ASM activity is a common feature used to differentiate NPA from other subtypes for counselling, prognostication, and the interpretation.

*SMPD1* gene mutations have been reported in many countries and ethnic groups, and mutation prevalence varies from one ethnic group to another [21, 31, 33, 34, 36, 55]. In this study, we observed frequency differences in the same sites of SMPD1 protein between the East Asians in gnomAD and Han Chinese in the “Huabiao” project (Fig. 3C). In the gnomAD, the most common variant in East Asians is p.Lys189GlnfsTer4 (c.564dup), while p.Glu508Lys (c.1522G > A) is the most common one in Han Chinese. Besides, the high-frequency mutation sites of the *SMPD1* gene highly vary in different populations reported in the literature, such as Ashkenazi Jews, Italians, Spanish, Turks, Chinese, and Dutch. Moreover, p.F333SfsX52, p.L304P, and p.R498L are the most common *SMPD1* gene mutations among Ashkenazi Jews, which are more likely to cause NPA [60, 61]. This difference in the mutation frequency may also contribute to a massive difference in the prevalence of NPA and NPB in various ethnicities and cause different phenotypes. Globally, the most common mutation is p.Arg610del, which has been associated with NPB [34, 62]. Similarly, p.Arg610del is also dominant in our collected cases. In contrast, the most common mutations among Chinese patients are p.Arg3AlafsX76 and p.H284SfsX7 [34].

Furthermore, with the two databases, ClinVar and ANNOVAR, and a deep learning algorithm, the EVE model, to improve the reliability, we predict 21 unreported variants that could be pathogenic [63], which can provide new information to interpret the related variants in *SMPD1* gene testing for NPD. The comparison of two databases shows the frequency of variant sites of the *SMPD1* gene in the Chinese Han group is different from Huabiao to the east Asian in the gnomAD. It is believed that these high-risk mutations might lead to spontaneous abortion as the *SMPD1* gene expression is high in the CNS system during development. Only a few pathogenic variants have been found in the Sap B domain, in NPA, NPB or the intermediate subtypes. Ponting suggested that ASM’s Sap B domain was homologous to the Saposin B protein responsible for the lysosomal degradation of several sphingomyelinases and five other known molecules [64]. Although the necessity might be unclear [65], Saposin B protein could activate and boost the degradation of many glycolipids and glycerolipids, unlike its other family members of Saposin A, C, and D, which adopt the high specificity to some proteins [64, 65]. It implies that the changes in the Sap B domain seem to disrupt the non-peptide bonds connecting amino acids, such as disulfide bonds, which does not cause a complete loss of ASM function [22]. Thus, it safely infers that SapB deficiency does not lead to NPA/NPB. This notion correlates with other reports that the changes in this domain could lead to intermediate or non-neurological types of ASM deficiency [36, 66]. 66.67% of variants are found in the calcineurin-like phosphoesterase domain (from 255th to 462nd amino acid). Sphingomyelin degradation was included in this type of phosphodiesterase superfamily, demonstrating that the amino acid changes due to variants would impact the function of the ASM and finally lead to the severe phenotype, NPA or the milder one, NPB. Metallo-dependent phosphatase-like domain (202nd to 497th amino acid) found 80.95% of the variants. This domain is associated with metabolite damage control [67]. Hence, if the variants occur in these domains, it is highly likely to lead to LSD, even NPD [65].

Studies that comprehensively expounded pathways related to NPD are barely found. In the present results, within the data above, we infer that the following scenario could be the mechanisms underlying NPD. The expression profiles of *SMPD1* in cells and tissues in healthy people help explain the complex symptoms of NPD. We can further connect the clinical phenotypes to the mutation pattern based on the *SMPD1* expression profiles in fetal and adult tissues. ASM is an enzyme essential for neurodevelopment. Normally, the mutations in the catalytic domain of SMPD1 have severe pathogenic effects because the lost catalytic function of the enzyme can significantly decrease ASM activity, which causes the accumulation of sphingomyelin and other sphingolipids that are toxic at elevated and nonphysiological levels. The clinical manifestations include rapid progressive psychomotor deterioration, liver and spleen enlargement, respiratory disease, jaundice, and death within 3 years [29, 33, 68]. Liver and spleen enlargement could be a compensation mechanism for the body to sustain ASM activities. Considering that *SMPD1* is universally expressed in many different cell types and tissues, it is expected that the dysfunction of SMPD1 protein should have a significant impact on many tissues, indicating that symptoms of NPD should present in the whole body without much specificity. The phenotypes reported in NPD are consistent with short stature, osteoporosis, sea-blue histiocytosis, microcytic anaemia, and bone-marrow foam cells. The expression profile of *SMPD1* in various cell types during development and various adult tissues can help us comprehensively decipher the potentially affected cell types and tissues of *SMPD1* mutation, which might have been ignored clinically. In addition, according to its expression profile, *SMPD1* is highly expressed in the heart, pancreas, thyroid, and kidneys during fetal development, which indicates that the dysfunction of the *SMPD1* gene should have possibly influenced these organs and the related phenotypes such as renal involvement in NPD is rarely reported [69, 70]. Therefore, in clinical application, clinicians are recommended to conduct comprehensive examinations while diagnosing patients with potential NPD, paying attention to the pathological abnormalities of these organs, during fetal development hepatosplenomegaly, splenomegaly, and neurological abnormalities.

We further reasoned that potential pathogeny at a gene level could correlate with the types of mutations owing to the positive correlation between gene expression and protein expression in Fig. 6D. Severe mutations resulting from deletion or insertion and stop gain led to the premature termination of the synthesis of the polypeptide chain of the *SMPD1* gene, or mutated polypeptide chains produce enzymes without biological activity or barely active domains (finally aberrant development) [71]. In patients with NPB, a single missense mutation only changes an amino acid, resulting in defective ASM with partial catalytic activity. Therefore, the ASM activity of NPB is higher than that of NPA, which explains the perspective that patients with NPA/NPB have the same pathogenic mutated genes, but the clinical manifestations are quite different. The pathogenic mutations of the *SMPD1* gene are primarily found in compound heterozygotes; the phenotype-genotype association study is particularly complicated. Therefore, the gene expression profiles of the *SMPD1* in different cell types of fetal development and adult tissues could further clarify which cell types are response to the dysfunction of tissues and the symptoms of the disease, which is important resource for understanding the pathogenic mechanisms underlying NPA and NPB. The period from 2 weeks post-conception to early childhood is crucial for developing the brain and other CNS organs [72]. Large amounts of sphingomyelin are needed to be converted to develop non-CNS cells [72]. In the present study, the expression pattern of *SMPD1* (Fig. 4) in various cell types of fetal demonstrated that SMPD1 dysfunction should significantly affect the functions and development of the circulatory system (heart and kidneys) and nervous system, which are essential for the survival of the fetal and infants. The placenta, vital to support fetal growth, also presents a high *SMPD1* gene expression. The individual clinical symptoms strongly correlate with the severity of *SMPD1* mutations, as the mutations would result in the functional decrease or even loss of the ASM activity in those cell types. Once those mutations cause decreased or the forfeit ability of ASM results in the ASM substrates, the sphingomyelin, accumulation, which would negatively affect individual fetuses. Finally, the excessive amount of accumulated sphingomyelin might lead to NPD phenotypes at an early age (namely, Type A) or the NPB (the late-onset phenotype). Among 53 adult tissues, the expression of *SMPD1* is relatively high in the liver and the spleen (the liver ranked 13, and the spleen ranked 20, in Fig. 5), which also suggests high levels of sphingomyelin in both tissues. Individuals with low ASM activities might not be able to convert sphingomyelin timely; thus, patients with NPA and NPB are featured with progressive hepatosplenomegaly and other organ dysfunction [33, 56, 73].

Our study is the first to comprehensively elucidate the effects of *SMPD1* mutation on cell types and at the tissue level, which provides new insights into the genotype-phenotype association and can help in the precise diagnosis of NPD. Admittedly, our study has certain limitations; the number of cases included in this study is relatively small, which could influence the AUC results; more cases should improve the model’s performance. In this study, we fail to comprehensively detect the relationship between phenotypes and genotypes because of incomplete phenotype data from some reported cases. In addition, we found the area or ethnicity specificity to the frequency of the variants, but it should be noted that some mutations with population or area-specific prevalence could also result from the bias of case study and collection. However, we compared the frequency of the variants collected to the public databases, gnomAD and the results are consistent. For example, p.Arg610del are the most frequent variant in the documented ASMD patients (gnomAD databases) and the cases we collected. It would be essential to check the SMPD1 protein levels in different tissues and other protein expressions during SMPD1 secretion and genome-wide association studies [74] which could further explain the mechanism underlying ASMD.

## Conclusions

As a rare disease, symptoms of NPD are scattered in the whole body without much specificity. It is often misdiagnosed in different specialities. Many researchers have reported that patients with the same mutation site in a candidate pathogenetic gene always have different phenotypes; we also observe similar events in patients with NPD, indicating that other unidentified factors can contribute to the clinical manifestation. Therefore, caution should be taken when interpreting the effect of gene mutation on inherited diseases. It can be anticipated that with the whole genome sequence technology being gradually applied to the clinical diagnosis field, comprehensively deciphering the underlying mechanism for inherited disease will be a routine procedure with which the genetic factors and their interaction with diseases will be fully illustrated. It is also suggested that noninvasive prenatal testing with the whole genome sequence technology [74] could be incorporated into the national healthcare program that has reduced the prevalence of inherited diseases in China; thus, the prediction for pathogenicity will also be improved with the application of deep learning [75].

## Materials and methods

### Case collection

We searched PubMed using ‘Niemann-Pick disease’ and ‘mutation’ as keywords. We selected the data with a clear statement of the patient’s information (age, gender, nationality or ethnicity background etc.) and the corresponding types of NPD, mutation sites, clinical symptoms and/or ASM level.

### Data source

A dataset of GSE199194 [76] was downloaded from the Gene Expression Omnibus (GEO, http://www.ncbi.nlm.nih.gov/geo/). We obtained three mass spectrometry datasets of ASM protein expression, whose authors are Kushner et al. [40], Wang et al. [77] and Carlyle et al. [39] from the website Expression Atlas [78]. Proteins expression of ASM were identified from proteomicsDB (www.proteomicsdb.org), which contains a repository of human proteome information [79–81] .

### Cuttoff value to distinguish NPA and NPB based on ASM activity

The “pROC” package (version 0.2.3) in R (version 3.6.1, R Foundation for Statistical Computing, Vienna, Austria) was used to analyse ASM ratio data (the ratio of ASM activity in patients to the reference value of healthy people that the authors of the publications stated) to classify response groups and visualised by “ggplot2” R package. The predictive performance of each model was evaluated using the receiver operating characteristic curve (ROC) and the area under the curve (AUC). The t-test was used to test whether ASM activity has a difference between the two groups, with *p* < 0.05 considered significant.

### Pathogenicity annotation for mutations in the *SMPD1* gene

*SMPD1* has been confirmed to cause NPA and NPB. To date, many mutation sites have been detected in patients with NPD. To comprehensively identify pathogenic mutations in *SMPD1* that cause NPD, a pathogenicity analysis was conducted for all potential mutation sites in *SMPD1* genes with ANNOVAR (https://annovar.openbioinformatics.org/en) [82]. The annotated pathogenetic effect of each mutation in *SMPD1* was retrieved from ClinVar. PolyPhen-2 (http://genetics.bwh.harvard.edu/pph2/) was used to predict the pathogenic effect of missense on the protein. EVE scores for the *SMPD1* variants in proteins sequence were retrieved from the EVE model (https://evemodel.org/proteins/ASM_HUMAN) [30].

To comprehensively detect the mutation profile of *SMPD1*, we extracted the mutation frequency of *SMPD1* from the gnomAD (https://gnomad.broadinstitute.org/). The Han Chinese populations’ allele frequency of *SMPD1* was obtained from Huabiao (https://www.biosino.org/wepd).

### The expression pattern of the *SMPD1* gene in various cell types and tissues

The expression patterns of *SMPD1* in adult tissues were extracted from the GTEx portal (https://www.gtexportal.org/), and the expression patterns of *SMPD1* at the cell type level were downloaded from Descartes (https://descartes.brotmanbaty.org/). GTEx is a data resource and tissue bank used to investigate the relationship between genetic variation and gene expression in human tissues. The currently released platform includes genotype data from approximately 714 donors and 11,688 RNA-seq samples across 53 tissue sites. The Descartes database hosted the human gene expressions of over 4 million cells of 121 human tissues during fetal development. Fetal gene expressions of *SMPD1* in different cells were downloaded from the Gene Expression Omnibus platform (GSE156793).

### Differentially expressed genes between the treatment and the control group from the GSE199194

Empirical Analysis of Digital Gene Expression Data in R [83] is a differential expression screening method based on a negative binomial distribution generalised linear model, and here we used the R package “edgR” (version 3.34.0) for differential analysis to obtain differential expression genes between treated groups and controls.

### Functional analysis

We selected the top 50 DEGs with Log_2_FoldChange > 2 with FDR < 0.01 for gene set function enrichment analysis. We used Gene Ontology (GO) annotations of genes from the R package “org.Hs.eg.db” (version 3.1.0) as background, mapped genes to the background set, and performed enrichment analysis using the R package “clusterProfiler” [84] (version 3.14.3) to obtain the results of gene set enrichment. The minimum gene set was set to 5, and the maximum gene set to 5000; a FDR of< 0.01, were considered statistically significant.

### Statistical analysis

R software (version 3.6.1) was used to conduct all statistical analyses. *p* < 0.05 is seen as the significant difference.

## Supplementary Information

**Additional file 1:** **Supplementary Table S1.**

**Additional file 2:** **Supplementary Table S2.**

**Additional file 3:** **Supplementary Table S3.**

## Data Availability

All data are submitted within the paper.

## Abbreviations

ASM: Acid sphingomyelinase
CNS: Central nervous system
DEGs: Differentially expressed genes
GD: Gaucher’s disease
GO: Gene Ontology
KD: Krabbe disease
LSD: Lysosome storage disease
ML: Metachromatic Leukodystrophy
NPD: Niemann–Pick disease
NPA: NPD type A
NPB: NPD type B
SM: Sphingolipid sphingomyelin
